# Precision Electrochemical Micro-Machining of Molybdenum in Neutral Salt Solution Based on Electrochemical Analysis

**DOI:** 10.3390/mi15101191

**Published:** 2024-09-26

**Authors:** Yuqi Wu, Guoqian Wang, Moucun Yang, Yan Zhang

**Affiliations:** School of Mechanical and Power Engineering, Nanjing Tech University, Nanjing 211816, China

**Keywords:** electrochemical micro-machining (EEM), electrochemical analysis, micro-machining of molybdenum

## Abstract

Molybdenum is an important material in modern industry, widely used in extreme environments such as rocket engine nozzles and microelectrodes due to its high melting point, excellent mechanical properties, and thermal conductivity. However, as a difficult-to-machine metal, traditional machining methods struggle to achieve the desired microstructures in molybdenum. Electrochemical machining (ECM) offers unique advantages in manufacturing fine structures from hard-to-machine metals. Studies have shown that molybdenum exhibits a fast corrosion rate in alkaline or acidic solutions, posing significant environmental pressure. Therefore, this study investigates the electrochemical machining of molybdenum in neutral salt solutions to achieve high-precision microstructure fabrication. First, the polarization curves and electrochemical impedance spectroscopy (EIS) of molybdenum in NaNO3 solutions of varying concentrations were measured to determine its electrochemical reaction characteristics. The results demonstrate that molybdenum exhibits good electrochemical reactivity in NaNO3 solutions, leading to favorable surface erosion morphology. Subsequently, a mask electrochemical machining technique was employed to fabricate arrayed microstructures on the molybdenum surface. To minimize interference between factors, an orthogonal experiment was used to optimize the parameter combination, determining the optimal machining process parameters. Under these optimal conditions, an array of micro-groove structures was successfully fabricated with an average groove width of 110 μm, a depth-to-width ratio of 0.21, an aspect ratio of 9000, and a groove width error of less than 5 μm.

## 1. Introduction

Molybdenum is an important industrial material [1] that is often used as an alloying element in metallurgical engineering to improve the mechanical properties and corrosion resistance of alloys [2]. It is also considered a strategic material due to its high melting point, ability to maintain high strength, low expansion coefficient [3], and good electrical and thermal conductivity under extreme conditions. It is widely used in various extreme conditions, such as microelectrodes, emitter tips, heating elements, and rocket engine nozzles [4]. However, its excellent physical and mechanical properties make it difficult for molybdenum to be machined by traditional metal cutting and obtain fine structures with ideal precision. Compared with metal cutting, electrochemical machining technology has outstanding merits as it has no physical and mechanical properties, no physical contact, no residual stress after processing, and no recast layer, with excellent application prospects in the manufacture of molybdenum [5,6,7].

In electrochemical machining, the dissolution characteristics of the material in the electrolyte directly determine the machining parameters and final machining accuracy. Electrochemical corrosion experiments have been conducted to understand the corrosion and dissolution characteristics of molybdenum in different types and concentrations of solutions. Badawy studied the electrochemical corrosion of molybdenum in alkaline solution [8], finding that the dissolution rate increased with the concentration of the solution. Misirlioglu studied the corrosion behavior of molybdenum in acidic solutions [9], while Scheider examined the dissolution behavior of molybdenum in both acidic (pH = 1) and alkaline (pH = 12) sodium nitrate solutions, confirming the existence of anodic oxygen evolution [10]. Most previous studies have been carried out in alkaline and acidic solutions, but such solutions lead to significant environmental concerns in application. As a more environmentally friendly solution, a neutral electrolyte is the main choice for electrochemical machining. However, there are few studies on the corrosion behavior of molybdenum in neutral solution.

In previous studies, Badawi and Al-Kharafi found that the surface of molybdenum in solution was always covered by a layer of passivation film, which was less stable in alkaline solution, in which soluble substances appeared (HMoO_4_^−^ and MoO_4_^2−^) [11]. This is because hydroxyl ions (OH^−^) play a major role in dissolving the passivation film on the surface of molybdenum. Therefore, in theory, molybdenum and its passivation film can also be dissolved in a neutral solution, as the pulse voltage will produce enough OH^−^ in a neutral solution during electrochemical processing [12,13]. Thus, the generated OH^−^ ions can dissolve the passivation film on the surface of molybdenum in a neutral solution.

Measuring polarization curves and electrochemical impedance spectroscopy (EIS) are common and reliable research methods used to understand the dissolution characteristics of metals in different solutions. Hu studied the effect of chloride ion concentration on the electrochemical corrosion behavior of molybdenum alloy by means of polarization curves and scanning electron microscopy (SEM). With the increase in concentration, the corrosion rate first increased and then decreased [14]. Hu then analyzed and discussed the Cl^−^ corrosion behavior of pure zirconium at different concentrations by electrochemical testing and numerical fitting [15]. Gao proved the feasibility of tungsten processing in a neutral solution by measuring polarization curves, among other research methods [16].

To study the electrochemical corrosion characteristics of molybdenum in neutral solution, in this work, the potential polarization curves and AC impedance spectra of molybdenum in various neutral solutions using an electrochemical workstation. The electrochemical behavior of different concentrations of neutral solution was analyzed. Electrochemical test results showed that molybdenum in alkaline solution was indeed more susceptible to corrosion. However, molybdenum also showed a notable corrosion tendency in neutral solution. Especially in NaNO_3_ solution, molybdenum not only demonstrated good electrolytic reaction efficiency but also good corrosion morphology. Subsequently, electrochemical micro-machining experiments of molybdenum with a micro-groove structure were carried out in different concentrations of NaNO_3_ solution. The optimal parameter combination of concentration, processing voltage, and processing time was obtained by orthogonal experiments. High-precision machining of the micro-grooves array was realized under these optimized parameters.

## 2. Potential Polarization Curve Measurement Experiment

### 2.1. Preparation of Pure Molybdenum Electrochemical Sample

To ensure the accuracy of the experiment, a cubic molybdenum block with dimensions of 10 mm × 10 mm × 10 mm was polished stepwise with 600-, 800-, 1200-, 2000-, and 3000-mesh sandpaper before the experiment and then polished by a polishing machine. This polishing was performed to remove the oxide layer and scratches on the surface, followed by cleaning with deionized water and acetone. The test device used a traditional three-electrode system, with platinum as the auxiliary electrode, a saturated calomel electrode (SCE) as the reference electrode, and the test sample as the working electrode. The test conditions are shown in Table 1 and Table 2. The open circuit potential, EIS, and polarization curves of molybdenum in these solutions were measured using the CHI660e electrochemical workstation. After the test, the polarization curves of molybdenum in different solutions were drawn.

**Figure 1:**
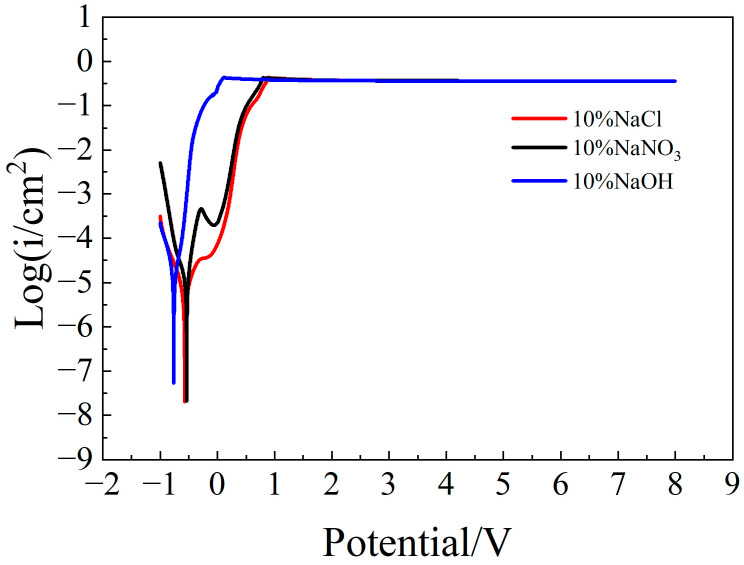
Polarization curves of molybdenum in different solutions.

Figure 1 shows the polarization curves of the molybdenum sample in three different solutions. It was obvious that whether in an alkaline solution or in a neutral solution, after reaching a certain potential, the polarization curves reached a constant level and were at the same level of height. However, the electrolytic reaction characteristics of molybdenum in alkaline solution and neutral solution were also significantly different. The active potential of molybdenum in the alkaline solution was lower than that in the neutral solution, and there was an obvious passivation region in the neutral solution. The main reason for the above phenomenon was the OH^−^ ion of the solution. According to Equation (1), molybdenum oxide can be dissolved by reacting with OH^−^ [11]. Therefore, molybdenum in alkaline solution showed stronger electrolytic reactivity, and there was no passivation region. In contrast, in the neutral solution, the amount of OH^−^ was very small, the oxide film was difficult to dissolve naturally, the reaction resistance was large, and the electrolysis reaction rate was slow. However, as the test progressed, under the action of the electric field, the concentration of OH^−^ ions in the solution increased, the dissolution of the oxide film accelerated, the current density gradually increased, and the electrolysis reaction rate also accelerated simultaneously. In addition, molybdenum exhibited an obvious passivation region in the NaNO_3_ solution. Molybdenum typically forms a stable passive layer in neutral regions. With the involvement of nitrate ions, this passive layer may be further reinforced through oxidation reactions, leading to a distinct passivation zone. As the potential increased, the passivation film dissolved or peeled off, and the dissolution rate of the anode metal began to increase. Through the polarization curves of molybdenum in three different solutions, it was found that molybdenum can undergo an electrolytic reaction in a neutral solution. In particular, there was an obvious passivation phenomenon in the NaNO_3_ solution, which is beneficial to improving the localization accuracy of electrochemical machining. Therefore, the passivation characteristics of molybdenum in NaNO_3_ solution were further studied, and the polarization curves of molybdenum in different concentrations of NaNO_3_ solution were measured.

(1) $${MoO}_{3}+{2OH}^{-}\to {HMoO}_{4}^{-}+H_{2}O+e^{-}$$

**Figure 2:**
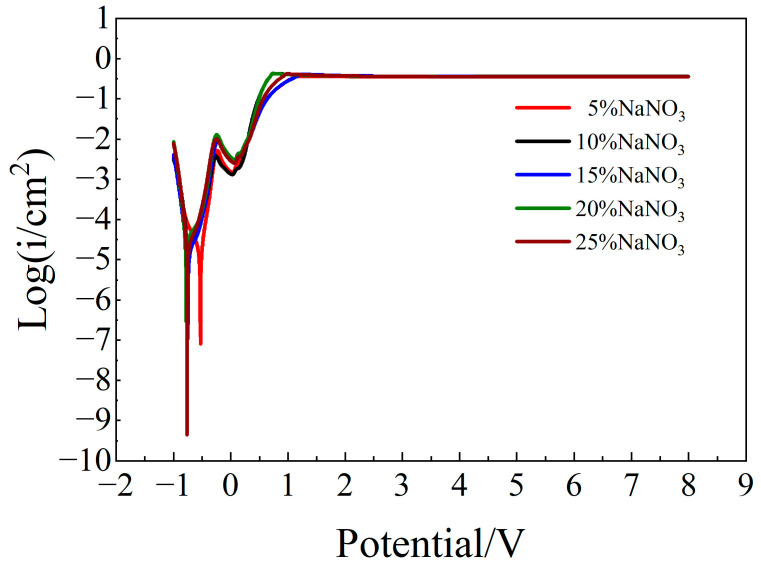
Polarization curves of molybdenum in different concentrations of NaNO_3_ solution.

Figure 2 shows the polarization curves of molybdenum in different concentrations of NaNO_3_ solution. The corrosion potential at a 5% concentration is significantly higher than that at other concentrations. The corrosion potentials at other concentrations are relatively close, indicating that corrosion is more likely to occur in 10–25% NaNO_3_ solutions. The passivation region of molybdenum became smaller with the increase of NaNO_3_ concentration, and there were some differences in the corrosion rate of the polarization curves. However, there were no obvious differences in electrolysis reaction characteristics in different concentrations of NaNO_3_ solution, and these reactions eventually reached a constant rate. In the range of 5~20% NaNO_3_ solution concentration, the electrolytic reaction rate of molybdenum gradually increased with the increase of solution concentration, especially for concentrations of 10% and 20%, showing strong electrolytic reaction characteristics. However, when the concentration of NaNO_3_ solution increased to 25%, the electrolysis reaction rate decreased. With the increase of NaNO_3_ solution concentration, the electrolysis reaction rate of molybdenum first increased and then decreased. This was due to the increase in the concentration of the solution and the active ions therein, which enhanced conductivity and turned the surface film into a porous passivation film. When the surface protection of the porous passivation film was reduced, the metal became in direct contact with the corrosive medium, and the corrosion rate was accelerated. However, after reaching the critical concentration, the porous passivation film tended to stabilize, and the corrosion rate decreased. This result is consistent with the EIS measurements described in the following section. In terms of the *R*_2_ value of the oxide film fitted by the AC impedance spectrum, the values of the oxide film in the 10% and 20% NaNO_3_ solutions were lower, and those in other solution concentrations were higher. Therefore, the corrosion resistance of the passive film of molybdenum was poor in the 10% and 20% solutions. In short, differences in the concentration of the NaNO_3_ solution led to differences in the passivation film, which in turn affected the electrolysis reaction characteristics of molybdenum in different concentrations of solution. Among them, the electrolysis reaction characteristics of molybdenum were stronger in the 10% and 20% NaNO_3_ solutions.

### 2.2. Corrosion Surface Microstructure of Molybdenum

**Figure 3:**
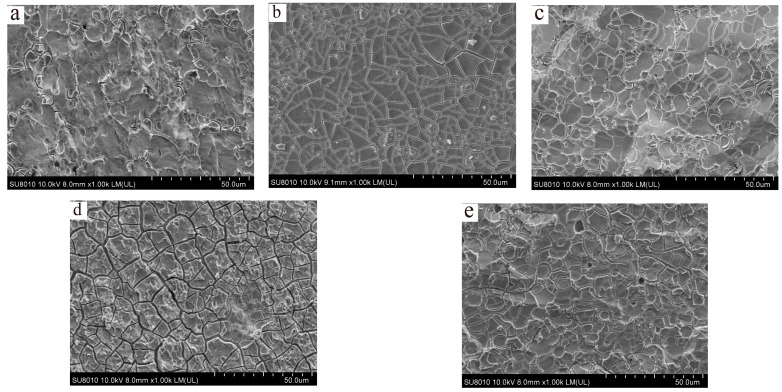
Scanning electron microscopy images of surface morphology of molybdenum electrolyzed in five different concentrations of NaNO_3_ solution after polarization curve testing: (**a**) 5% NaNO_3_ solution, (**b**) 10% NaNO_3_ solution, (**c**) 15% NaNO_3_ solution, (**d**) 20% NaNO_3_ solution, and (**e**) 25% NaNO_3_ solution.

The polarization curves showed that molybdenum can be electrolytically reacted in NaNO_3_ solution. However, for electrochemical micro-machining, the surface morphology of the anode is also a significant indicator while ensuring a certain electrolysis reaction rate. Therefore, the surface morphology of molybdenum was observed via SEM after the polarization curve tests. Figure 3 presents SEM images of the molybdenum electrolytic surface after electrolysis in different concentrations of NaNO_3_ solution, with different corrosion morphologies observed for different NaNO_3_ concentrations. In the 5% NaNO_3_ solution, the metal surface exhibited an uneven appearance, there was a tendency for corrosion, irregular corrosion cracks appeared, the degree of corrosion was still relatively shallow, and the electrolytic reaction rate was slow. In 10% NaNO_3_ solution, after the metal was electrolyzed, the surface was divided into reticular cracks, which were corroded downward along the cracks to form a corrosion groove, and the metal surface was divided into multiple areas. In the 15% NaNO_3_ solution, the metal surface also showed reticular cracks after electrolysis, and the cracks were small, similar to those in the 10% NaNO_3_ solution. However, in the 20% NaNO_3_ solution, after the metal surface was electrolyzed, the same network cracks appeared as in the previous 10% solution. However, compared with the surface after electrolysis in 10% NaNO_3_ solution, the surface cracks in 20% NaNO_3_ solution were more obvious, and their depth and width were larger, indicating that the reaction in 20% NaNO_3_ solution was more intense. However, in 25% NaNO_3_ solution, the surface of molybdenum was relatively flat after electrolysis, and the degree of electrolytic reaction was decreased. The SEM imaging showed that no matter which concentration of neutral solution was used, metastable pitting could occur on the metal surface. Therefore, ions could reach the metal surface through the passivation film, leading to pitting, the degree of which depended on the concentration of NaNO_3_ solution used. The electrolysis reaction characteristics of the surface morphology reaction after molybdenum electrolysis were consistent with the polarization curve test results. As the concentration of NaNO_3_ solution increased, the electrolysis reaction rate of molybdenum first increased and then decreased. Among them, in 10% NaNO_3_ solution, molybdenum not only maintained a better electrolysis reaction rate, but an ideal surface morphology was obtained as well.

### 2.3. EIS of Pure Molybdenum

Electrochemical impedance spectroscopy (EIS) experiments were conducted to analyze the electrolytic reaction mechanism of molybdenum in different concentrations of NaNO_3_ solution further. EIS can effectively characterize the characteristics of the electrochemical system [17] and analyze the effects of electrode kinetics and anode passivation film on the electrolytic reaction [18]. According to the Nyquist diagram, the interface characteristics between the oxide film and the negative electrode of the anode material can be analyzed. When a semi-circular structure is present in the high-frequency region, it indicates the capacitance response of the oxide film. The diameter of the semicircle in the high-frequency region is related to the oxide film, where the oxide film resistance on the surface of the sample increases with the increase of the diameter of the semicircle in the high-frequency region [19]. To understand the effect of the anodic oxide film on corrosion resistance in different solutions, EIS curves of molybdenum in NaCl solutions, NaOH solution, and NaNO_3_ solution were obtained, and changes in electrochemical characteristics were analyzed.

**Figure 4:**
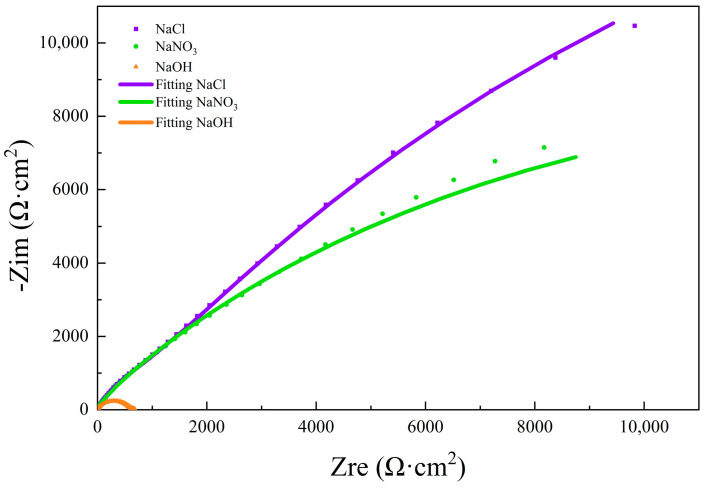
Nyquist plots of molybdenum in three different solutions.

Figure 4 shows the EIS results of pure molybdenum in three different solutions in the form of Nyquist plots. Three kinds of solutions showed an incomplete semicircle, representing the capacitive response of the surface oxide film. It can be seen that the radius in the 10% NaCl solution was the largest, indicating the highest oxide film resistance. The radius in 10% NaOH solution was the smallest, indicating the lowest oxide film resistance. Therefore, the corrosion resistance of molybdenum in 10% NaNO_3_ solution was the lowest, best facilitating the electrolytic reaction. These results are similar to the electrochemical corrosion characteristics of the polarization curves of molybdenum in three different solutions measured in Figure 1. Pure molybdenum showed a certain tendency for electrolytic reaction in a neutral solution. In 10% NaNO_3_ solution, the corrosion resistance of pure molybdenum was weakest, and it was easiest to carry out electrolytic processing. Subsequently, EIS tests of molybdenum in different concentrations of NaNO_3_ solution were carried out.

#### 2.3.1. EIS in NaNO_3_ Solutions with Different Concentrations

**Figure 5:**
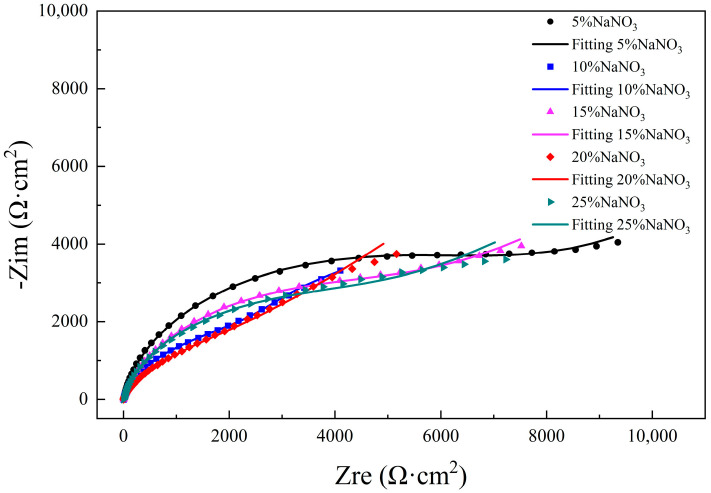
Nyquist plots of molybdenum in different concentrations of NaNO_3_ solution.

Figure 5 shows the electrochemical impedance spectroscopy of different concentrations of NaNO_3_ solution. All NaNO_3_ solutions with different concentrations exhibited incomplete semicircles, indicating a capacitive response. With the increase in concentration, the radius of the arc in the high-frequency region decreased; that is, the corrosion rate of molybdenum in the high-concentration NaNO_3_ solution increased, and the corrosion resistance decreased. However, a diffusion tail appeared in the low-frequency range of all NaNO_3_ solutions, which was due to the diffusion phenomenon caused by the corrosive medium passing through the film, indicating that the oxide film formed in NaNO_3_ solution had poor protection and was easily electrochemically processed.

**Figure 6:**
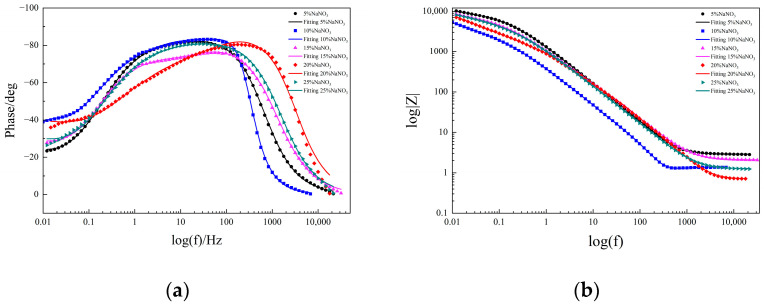
Bode diagrams of molybdenum in different concentrations of NaNO_3_ solution: (**a**) Bode phase angle diagram; (**b**) Bode amplitude diagram.

Figure 6 presents a Bode diagram of a molybdenum sample in different concentrations of NaNO_3_ solution. In Bode diagrams, the maximum phase angle is often used to reflect the corrosion resistance of the material in the solution. Typically, the maximum phase angle refers to the maximum phase difference between the current and the potential at a low frequency. The larger the maximum phase angle, the weaker the electrochemical activity and the stronger the corrosion resistance [20]. As seen in Figure 6a, the highest phase angle in the 10–20% NaNO_3_ solutions was lower at low frequencies, which indicates that the corrosion resistance of molybdenum was poor in these solutions. As seen in the Bode diagram in Figure 6b, the impedance modulus |Z| of pure molybdenum showed a downward trend with the increase of frequency in different concentrations of NaNO_3_ solution. In the Bode diagram of impedance, the larger the impedance amplitude, the stronger the corrosion resistance [21,22]. As seen in Figure 6, the impedance amplitude was lowest in the 10% NaNO_3_ solution, which indicated that the corrosion resistance of molybdenum was weakest, making it easier to carry out electrolytic processing.

#### 2.3.2. Fitting of EIS Values

**Figure 7:**
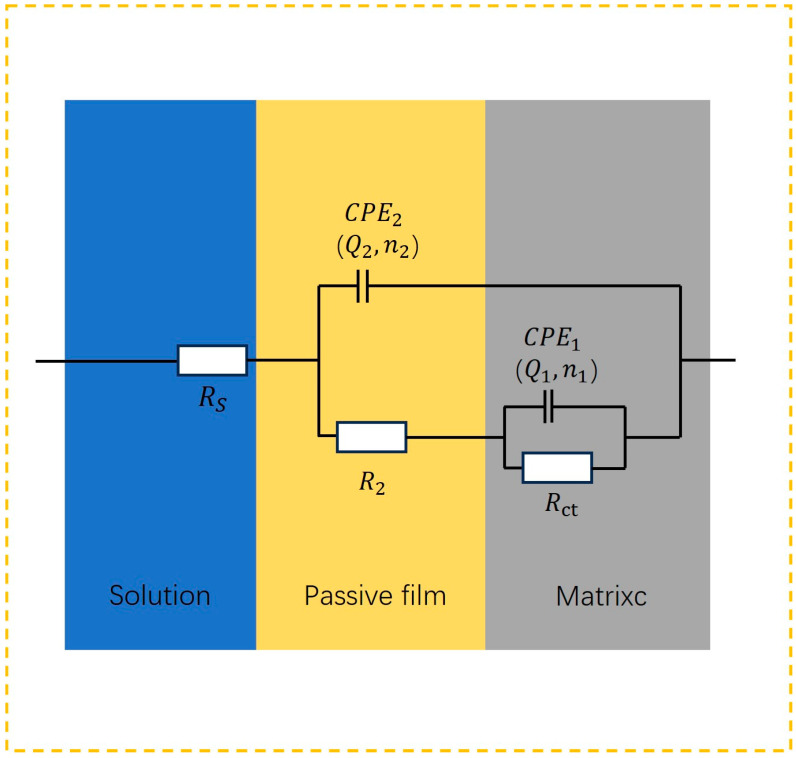
EIS fitting circuit diagram.

This is shown in the Nyquist diagram in Figure 5, where the molybdenum exhibited an incomplete semicircle in different concentrations of NaNO_3_ solution, indicating that there were multiple time constants. The Bode diagram in Figure 6 also showed that there were multiple peak phase angles in the intermediate-frequency region, which indicated that there were multiple time constants [23,24]. Some ions are chemically inert. These ions do not participate in chemical reactions and only change the charge distribution. They are often expressed using an electric double layer. Therefore, *CPE* is used to replace the ideal capacitance when non-ideal capacitance occurs [25]. *CPE* is defined as follows:

(2) $$Z_{CPE}={\left[{Q(i\omega )}^{n}\right]}^{-1}$$

where *Q* is a constant, ω is the angular frequency, *i* is the imaginary number, and *n* is the index of *CPE*, which is the standard to measure the roughness of the surface. The closer *n* is to 1, the smoother the surface is [26]. The *CPE* index is an empirical quantity commonly used to describe various physical phenomena, such as non-ideal capacitance behavior caused by surface inhomogeneity. Therefore, based on this information, an equivalent circuit model of series-parallel multiple time constants is used to model the impedance response of molybdenum in NaNO_3_ solution with different concentrations, as shown in Figure 7. In this model, *R*_s_ is the resistance of the electrolyte, *R*_2_ is the resistance of the oxide film, *CPE*_2_ is the capacitance of the oxide film, *R*_ct_ is the charge transfer resistance of the corrosion process, *CPE*_1_ is a double-layer capacitor, and *W*_o_ is the Warburg impedance, which is responsible for the diffusion process of the corrosive medium on the metal oxide film [27]. The circuit diagram shown in Figure 7 is used to model the impedance response of molybdenum in NaNO_3_ solution with different concentrations [28].

The fitting results are shown in Table 3. With the increase in concentration, the corrosion resistance of the oxide film of pure molybdenum in NaNO_3_ solution changed. The fitted value of *R*_2_ was lowest in 10% NaNO_3_ solution, which indicated that the corrosion resistance of the oxide film was poor and the structure of the oxide film was loose. *R*_ct_ represents the transfer charge resistance, which is an important parameter in the electrochemical reaction process. *R*_ct_ reflects the kinetics of charge transfer during corrosion, which is inversely proportional to the corrosion rate. As seen in Table 3, with the increase of NaNO_3_ solution concentration, the value of *R*_ct_ shows a trend of decreasing first, then increasing, and finally decreasing. The corrosion rate in 10% and 20% NaNO_3_ solutions was obviously faster than that in other solutions, and the corrosion resistance of metal molybdenum was worse. These results are consistent with the previous polarization curve measurement results, where it is easier to perform electrochemical micro-machining in NaNO_3_ solution with a concentration of 10% to 20%.

## 3. Electrochemical Micro-Machining Experiment of Molybdenum Micro-Groove Array Structure

**Figure 8:**
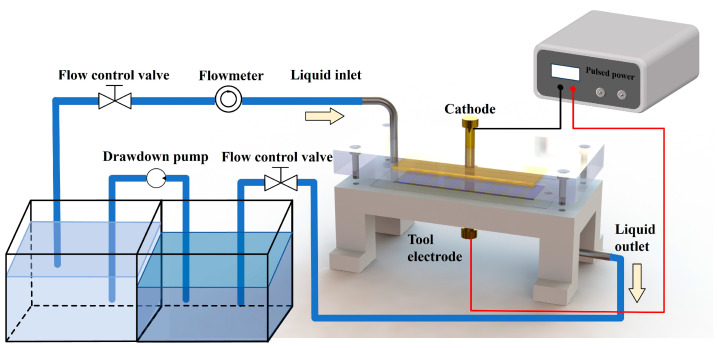
Principle diagram of electrochemical micro-machining (EMM).

Based on the previous polarization curve measurements, SEM image observation, and EIS testing of molybdenum in different concentrations of NaNO_3_ solution, our results showed that molybdenum can be electrochemically processed in NaNO_3_ solution. The electrolytic processability of pure molybdenum in NaNO_3_ solution was then studied experimentally. Mask electrochemical micro-machining was used in this experiment, whose basic processing principle is shown in Figure 8. The anode processing area is limited by an insulating mask, and the mask structure is photolithographed to the metal surface. A clamp is used to ensure the close coordination of the anode workpiece and the cathode, and a certain gap is maintained to ensure that the electrolyte can pass through. After energization, the surface of the workpiece is etched, and circulation of the electrolyte removes the electrolytic product to prevent its aggregation [29,30,31,32]. In the experiment, pure molybdenum sheets with a length of 30 mm, width of 10 mm, and thickness of 1 mm were selected, and different concentrations of NaNO_3_ solution were used as the electrolyte. A mask plate with a micro-groove width of 50 μm was prepared by lithography.

Based on the previously mentioned polarization curves and EIS tests, it was found that different concentrations of NaNO_3_ solution had a certain effect on the electrolysis reaction of pure molybdenum. At the same time, In the preliminary experiments, it was found that different voltages lead to variations in micro-groove width, while the length of processing time affects the depth of the micro-grooves. Therefore, the main parameters in mask electrochemical micro-machining include processing voltage and processing time. Due to the mutual influence between various factors, the accuracy of single-factor experimental processing results is not enough. Therefore, an orthogonal experiment was used to optimize the processing parameters. Five variables were selected as the horizons of the orthogonal experiment for each processing parameter, and an orthogonal experiment of three factors and five horizons was designed [33,34,35,36]. The specific values of each factor and horizon are shown in Table 4.

**Figure 9:**
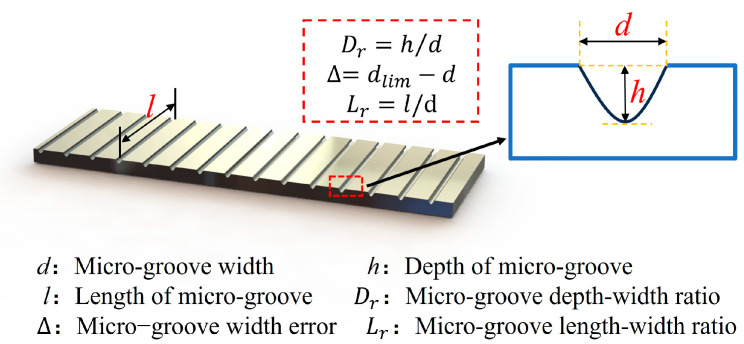
Measurement index diagram of micro-grooves array structure.

The basic structure of the array of micro-grooves processed in the experiment is shown in Figure 9. According to the machining accuracy requirements, the micro-groove depth-width ratio *D*_r_ and the micro-groove width error *∆* were used as the assessment indicators of the orthogonal test. The combination of all factors and levels of the orthogonal test are shown in Table 5. At the end of the experiment, a 3D profilometer (Keyence VR5000, Keyence, Japan) was used to measure the depth-width ratio and width error of micro-grooves, and then the measured orthogonal experimental data were analyzed.

**Figure 10:**
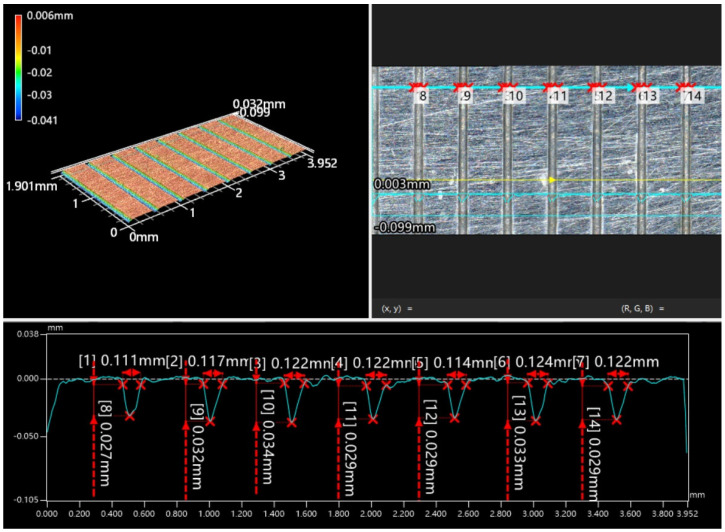
Measurement example of the experimental workpiece.

The average value of the test indexes of different processing factors at the same horizon is represented by *K*_i_ (*i* represents the horizon), and the range value under each factor is calculated. The larger the range, the greater the influence of the factor on the assessment index, and the more important the factor is to the assessment index. The workpiece after the experiment was measured with a 3D profilometer (Keyence VR5000). Due to the large number of workpieces, it cannot be shown one by one. Take the measurement results of the 20th group of experimental workpieces as an example. As shown in Figure 10, the groove width and groove depth at different positions of the micro-grooves array were measured, and the micro-grooves depth-width ratio *D*_r_ and micro-groove width error *∆* were calculated.

Table 6 presents the orthogonal analysis results with the depth-width ratio *D*_r_ as the evaluation index. The micro-groove structure was characterized by a semi-circular shape, so the larger the depth-width ratio, the better the parameters. From the range of the horizontal average of the depth-width ratio *D*_r_ in Table 6 under various factors, the key order of each factor of the index was electrolyte concentration > processing time > voltage. In other words, the effect of electrolyte concentration on the depth-width ratio of the microgroove structure was the strongest among all factors. According to the test indexes at each level, the optimal combination was an electrolyte concentration of 15%, a processing time of 60 s, and a voltage of 15 V (B3C5A2).

Table 7 shows the orthogonal analysis results with the groove width error *∆* as the evaluation index. The array micro-grooves were processed by EMM, so the smaller the groove width error, the better. From the range of the horizontal average value of the groove width error *∆* in Table 7 under various factors, the key order of this index was voltage > processing time > electrolyte concentration. In other words, voltage had the largest influence on groove width error. According to the test indexes at each level, the optimal combination was a voltage of 10 V, a processing time of 60 S, and an electrolyte concentration of 10% (A1C5B2).

**Figure 11:**
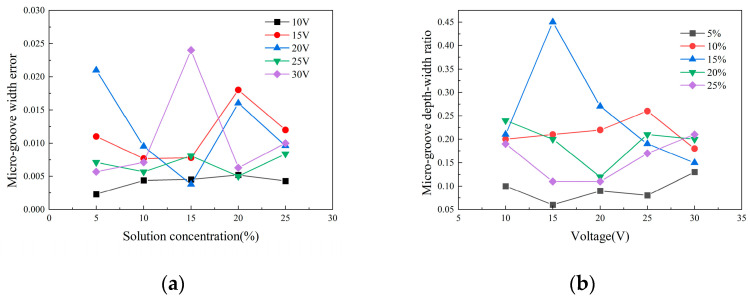
Optimization of micro-groove processing results: (**a**) Comparison of micro-groove error of molybdenum processed with different concentrations of NaNO_3_ solution under the same voltage; (**b**) Comparison of microgroove depth-width ratio of molybdenum processed with different voltages under the same concentration of NaNO_3_ solution.

The above optimization results used depth-width ratio and groove width error as indicators, so it is not difficult to find that the optimal combination under different test assessment indicators was not the same, which required further analysis. Based on the previous optimization results, using the depth-width ratio and groove width error as key indicators, it was determined that a processing time of 60 s was the optimal processing time, so processing time was no longer analyzed as an influencing factor. The optimization results on the micro-groove depth-width ratio *D*_r_ and groove width error *∆* were not the same. In Figure 11, Figure 11a is a comparison of micro-groove error of molybdenum processed with different concentrations of NaNO_3_ solution under the same voltage; Figure 11b is a comparison of microgroove depth-width ratio of molybdenum processed with different voltages under the same concentration of NaNO_3_ solution. As seen in Figure 11a, at an electrolyte concentration of 10% (B2), the groove width error *∆* at each voltage was more clustered than that under other electrolyte concentrations. Therefore, the processing at this concentration was more stable, and the groove width error *∆* fluctuated the least. Therefore, an electrolyte concentration of 10% (B2) was selected as the optimal result. As seen in Figure 11b, the depth-width ratio *D_r_* was less affected by voltage at electrolyte concentrations of 5% (B1) and 10% (B2). However, the depth-width ratio *D_r_* was significantly higher at an electrolyte concentration of 10% (B2) than at 5% (B1). In summary, 10% NaNO_3_ (B2) was selected as the optimal result for electrolyte concentration.

For the processing voltage, Figure 11a shows that when the processing voltage was 10 V (A1), the fluctuation of the groove width error was the smallest. However, the distribution of the depth-width ratio *D*_r_ had no obvious regularity, and the processing voltage could not be directly optimized. Therefore, on the basis of the existing optimization results, processing experiments of two sets of parameter combinations—a voltage of 10 V, an electrolyte concentration of 10%, and a processing time of 60 s; and a voltage of 15 V, an electrolyte concentration of 10%, and a processing time of 60 s were carried out, so as to further analyze and optimize the processing voltage.

**Figure 12:**
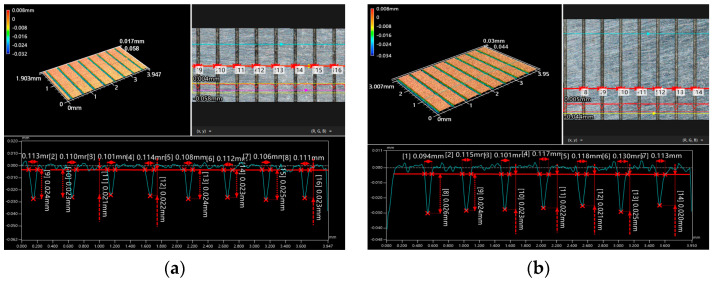
The 3D morphology of molybdenum surface micro-groove array under different processing parameters: (**a**) voltage of 10 V, electrolyte concentration of 10%, and processing time of 60 s; (**b**) voltage of 15 V, electrolyte concentration of 10%, and processing time of 60 s.

Through the previous optimization scheme, the optimal combination of electrolyte concentration and processing time was determined. Therefore, electrochemical micro-machining experiments on molybdenum were conducted at processing voltages of 10 V and 15 V while maintaining the same electrolyte concentration and processing time, with the results shown in Figure 12. By measuring these two sets of micro-grooves, it was found that when the processing voltage was 10 V, the groove width error was 5 μm, and the depth-width ratio was 0.21. When the processing voltage was 15 V, the groove width error increased to 18 μm, while the depth-width ratio was 0.20. The results indicated that the depth-width ratio did not vary significantly under different voltages, but the micro-groove width error was smaller at a processing voltage of 10 V. When comparing the surface morphology, as seen in Figure 12a,b, under a processing voltage of 10 V, the micro-groove boundaries were clearer and sharper, and the micro-groove depth was more uniform. These results demonstrated that when the processing voltage was 10 V, the processing stability was better, and the processing accuracy was higher. Therefore, the optimal parameter combination for array micro-grooves in electrochemical machining was a processing voltage of 10 V, an electrolyte concentration of 10%, and a processing time of 60 s (A1B2C5). Under these parameters, the array micro-grooves were successfully processed, exhibiting a depth-width ratio of 0.21, a width error of less than 5 μm, a length-width ratio of 9000, and relatively flat surfaces with high machining accuracy. Thus, precision electrochemical machining of molybdenum surface micro-structures can be achieved using NaNO_3_ solution.

## 4. Conclusions

Molybdenum is an important industrial metal material that is widely used in various fields. Neutral salt solutions have good application prospects for realizing the precision machining of fine-structure molybdenum. In this paper, the electrolysis reaction characteristics of molybdenum in different electrolyte solutions were analyzed by means of polarization curves and EIS. It was confirmed that molybdenum can be electrolyzed in NaNO_3_ solution. Subsequently, an orthogonal experiment of EMM was carried out, and a micron-scale micro-groove array structure was processed on the molybdenum surface. By measuring and analyzing the processing results, the optimal combination of electrolytic processing parameters was determined. This research is expected to further explore the deposition of free shapes of molybdenum metal and facilitate practical device applications in industries such as semiconductors and aerospace. The specific research conclusions are as follows:

1. The polarization curves of molybdenum in different concentrations of NaNO_3_ solution show passivation characteristics that are not found in alkaline solution. NaNO_3_ solution was found to readily cause a dense oxide film to form on the surface of molybdenum. At the same time, with the increase in concentration, the dissolution rate of passivation film first increases and then decreases. Thus, molybdenum can be electrolytically reacted in a certain concentration of NaNO_3_ solution with good localization accuracy.
2. By analyzing the EIS of molybdenum in different concentrations of NaNO_3_ solution and its surface morphology after electrochemical testing, it was found that the impedance of the molybdenum surface oxide film changed with the increase of NaNO_3_ solution concentration. In 5–10% NaNO_3_ solution, the impedance of the oxide film on the surface of molybdenum decreased with the increase of concentration, the electrolysis reaction characteristics were enhanced, and the electrolysis reaction rate increased. However, after increasing to a certain concentration, the resistance of the oxide film on the surface of molybdenum increased, and the electrolysis reaction rate decreased. After comprehensive analysis, it was found that the electrolysis reaction rate of molybdenum in 10–20% NaNO_3_ solution was faster, and the surface morphology after electrolysis was better.
3. EMM processed a micro-groove structure on the surface of the molybdenum. Design an orthogonal experiment, and the optimal parameter combination was determined by using microgroove depth-width ratio and micro-groove error as indicators. During the optimization process, it was found that the depth of the micro-groove increased with the increase of processing time. In 10% NaNO_3_ solution, it was found that the fluctuation of groove-width error was the smallest, and the depth-width ratio of the microgrooves was the largest. When the processing time and electrolyte concentration were held constant, the processing effect at a voltage of 10 V was highest. Therefore, according to the orthogonal test, the optimal parameters were obtained, comprising a NaNO_3_ solution concentration of 10%, a voltage of 10 V, and a processing time of 60 s. Under these parameters, the depth-width ratio of the array microgrooves was 0.21; the microgroove width error *∆* was less than 5 μm and a length–width ratio of 9000. The experimental results showed that the precise electrochemical micro-machining of molybdenum surface micro-structures can be achieved by using a NaNO_3_ neutral salt solution.

## Figures and Tables

**Table 1:** Electrochemical test conditions and parameters for different solutions.

Electrolyte	NaCl	NaNO_3_	NaOH
Concentration	10%	10%	10%
Work electrode	Molybdenum (10 mm × 10 mm × 10 mm)		
Counter electrode	Platinum sheet		
Reference electrode	Saturated calomel electrode		
Potential range	−2 V to 8 V		
Scanning rate	0.01		

**Table 2:** Electrochemical test conditions and parameters for different concentrations of NaNO_3_ solution.

Electrolyte	NaNO_3_
Concentration	5%, 10%, 15%, 20%, 25%
Work electrode	Molybdenum (10 mm × 10 mm × 10 mm)
Counter electrode	Platinum sheet
Reference electrode	Saturated calomel electrode
Potential range	−2 V to 8 V
Scanning rate	0.01

**Table 3:** EIS fitting results.

Specimen Condition	5% NaNO_3_	10% NaNO_3_	15% NaNO_3_	20% NaNO_3_	25% NaNO_3_
*R*_s_	2.92	1.405	2.077	0.7082	1.244
*CPE*_1_	6.323 × 10^−5^	4.9868 × 10^−4^	8.47 × 10^−5^	2.24 × 10^−4^	2.5 × 10^−4^
*n*_1_	0.6584	0.824	0.7629	0.6266	0.28
*R*_2_	391.9	10.29	461.1	97.13	1022
*CPE*_2_	9.438 × 10^−5^	4.22 × 10^−5^	1.17 × 10^−4^	6.87 × 10^−5^	1.49 × 10^−4^
*n*_2_	0.9834	1	0.9249	1	0.934
*R*_ct_	7507	3169	5284	3471	5282
*W*	8.291 × 10^−4^	8.818 × 10^−4^	7.655 × 10^−4^	5.16 × 10^−4^	7.526 × 10^−4^
Chi-Squared	1.754 × 10^−4^	3.3525 × 10^−4^	1.727 × 10^−4^	2.20 × 10^−3^	4.05 × 10^−4^

**Table 4:** Orthogonal experiment with three factors and five horizons.

Processing Parameter		Horizon				
		1	2	3	4	5
A	Voltage (V)	10	15	20	25	30
B	Electrolyte concentration (%)	5	10	15	20	25
C	Processing time (S)	20	30	40	50	60

**Table 5:** Orthogonal experiment table and processing data results.

Group Number	Experimental Factor			Evaluation Index	
	A	B	C	Dr	*Δ*(*mm*)
1	1	1	1	0.10	0.0023
2	1	2	2	0.20	0.0044
3	1	3	3	0.21	0.00455
4	1	4	4	0.24	0.0052
5	1	5	5	0.19	0.0043
6	2	1	2	0.06	0.011
7	2	2	3	0.21	0.0077
8	2	3	4	0.45	0.0078
9	2	4	5	0.20	0.018
10	2	5	1	0.11	0.012
11	3	1	3	0.09	0.021
12	3	2	4	0.22	0.0095
13	3	3	5	0.27	0.0038
14	3	4	1	0.12	0.016
15	3	5	2	0.11	0.0096
16	4	1	4	0.08	0.0071
17	4	2	5	0.26	0.0057
18	4	3	1	0.19	0.0081
19	4	4	2	0.21	0.0050
20	4	5	3	0.17	0.0084
21	5	1	5	0.13	0.0057
22	5	2	1	0.18	0.0071
23	5	3	2	0.15	0.024
24	5	4	3	0.20	0.0063
25	5	5	4	0.21	0.010

**Table 6:** Orthogonal analysis of depth-width ratio *D*_r_.

Factor	A	B	C
*K*_1_	0.188	0.092	0.14
*K*_2_	0.206	0.214	0.146
*K*_3_	0.162	0.254	0.176
*K*_4_	0.182	0.194	0.194
*K*_5_	0.174	0.158	0.21
Range	0.044	0.162	0.07
Key ranking	B > C > A		
Optimal combination	A2	B3	C5

**Table 7:** Orthogonal analysis of groove width error *∆*.

Factor	A	B	C
*K*_1_	0.00432	0.00942	0.0091
*K*_2_	0.0113	0.00688	0.0108
*K*_3_	0.01198	0.00982	0.00976
*K*_4_	0.00686	0.0101	0.00792
*K*_5_	0.01062	0.00886	0.00792
Range	0.00766	0.00322	0.0033
Key ranking	A > C > B		
Optimal combination	A1	B2	C5

## Data Availability

The original contributions presented in the study are included in the article, further inquiries can be directed to the corresponding author.

## References

[1] Lasheen T.A., El-Ahmady M.E., Hassib H.B., Helal A.S. (2015). Molybdenum Metallurgy Review: Hydrometallurgical Routes to Recovery of Molybdenum from Ores and Mineral Raw Materials. Miner. Process. Extr. Met. Rev..

[2] Abbas Q., Binder L. (2011). The electrochemical dissolution of molybdenum in non-aqueous media. Int. J. Refract. Met. Hard Mater..

[3] Zhang D., Dong D., Xiong N., Dong Z., Ma Z. (2024). Research Progress in Dispersion Strengthened Molybdenum Alloys. Rare Met. Mater. Eng..

[4] Hu J., Zhang Y., Chen S., He S., Li N., Chen J. Inductively coupled plasma etching of bulk molybdenum. Proceedings of the IEEE 25th International Conference on Micro Electro Mechanical Systems (MEMS).

[5] Xu Z., Wang Y. (2021). Electrochemical machining of complex components of aero-engines: Developments, trends, and technological advances. Chin. J. Aeronaut..

[6] Goud M., Sharma A.K., Jawalkar C. (2016). A review on material removal mechanism in electrochemical discharge machining (ECDM) and possibilities to enhance the material removal rate. Precis. Eng..

[7] Qu N.S., Xu Z.Y. (2013). Improving machining accuracy of electrochemical machining blade by optimization of cathode feeding directions. Int. J. Adv. Manuf. Technol..

[8] Badawy W., Feky H., Helal N., Mohammed H. (2013). Cathodic hydrogen evolution on molybdenum in NaOH solutions. Int. J. Hydrogen Energy.

[9] Misirliogğlu Z., Aksüt A. (2002). Corrosion of Molybdenum in Aqueous Media. Corrosion.

[10] Schneider M., Šimůnková L., Michaelis A., Hoogsteen W. (2021). Electrochemical machining of molybdenum. Int. J. Refract. Met. Hard Mater..

[11] Badawy W.A., Al-Kharafi F.M. (1998). Corrosion and passivation behaviors of molybdenum in aqueous solutions of different pH. Electrochim. Acta.

[12] Leese R.J., Ivanov A. (2016). Electrochemical micromachining: An introduction. Adv. Mech. Eng..

[13] Leese R.J., Ivanov A. (2018). Electrochemical micromachining: Review of factors affecting the process applicability in micro-manufacturing. Proc. Inst. Mech. Eng. Part B.

[14] Hu P., Song R., Li X.-J., Deng J., Chen Z.-Y., Li Q.-W., Wang K.-S., Cao W.-C., Liu D.-X., Yu H.-L. (2017). Influence of concentrations of chloride ions on electrochemical corrosion behavior of titanium-zirconium-molybdenum alloy. J. Alloys Compd..

[15] He W., Luo S., Li N., He L., Yuan R., Xue Y. (2024). Electrochemical corrosion behavior of industrial pure zirconium in high Cl- containing solutions. Int. J. Electrochem. Sci..

[16] Gao C., Qu N. (2019). A Distinct Perception on Wire Electrochemical Micromachining of Pure Tungsten with Neutral Aqueous Solution. J. Electrochem. Soc..

[17] Ciucci F. (2019). Modeling electrochemical impedance spectroscopy. Curr. Opin. Electrochem..

[18] Zhang L.N., Ojo O.A. (2020). Corrosion behavior of wire arc additive manufactured Inconel 718 superalloy. J. Alloys Compd..

[19] Cairang W., Li T., Xue D., Yang H., Cheng P., Chen C., Sun Y., Zeng Y., Ding X., Sun J. (2021). Enhancement of the corrosion resistance of Molybdenum by La2O3 dispersion. Corros. Sci..

[20] Liu M., Zhu J.-N., Popovich V.A., Borisov E., Mol J.M.C., Gonzalez-Garcia Y. (2023). Corrosion and passive film characteristics of 3D-printed NiTi shape memory alloys in artificial saliva. Rare Met..

[21] Deng C.-M., Xia D.-H., Zhang R., Behnamian Y., Hu W., Birbilis N. (2023). On the localized corrosion of AA5083 in a simulated dynamic seawater/air interface—Part 2: Effects of wetting time. Corros. Sci..

[22] Zhang C., Huang L., Li S., Li K., Lu S., Li J. (2023). Improved corrosion resistance of laser melting deposited CoCrFeNi-series high-entropy alloys by Al addition. Corros. Sci..

[23] Rodríguez M.A., Carranza R.M. (2011). Properties of the Passive Film on Alloy 22 in Chloride Solutions Obtained by Electrochemical Impedance. J. Electrochem. Soc..

[24] Sun H., Wu X., Han E.-H. (2009). Effects of temperature on the protective property, structure and composition of the oxide film on Alloy 625. Corros. Sci..

[25] Yang H.J., Han D., Kim J., Kim Y.H., Bae J.H. (2022). Constant phase element affected by ion transport in nanoporous electrodes. J. Electroanal. Chem..

[26] Herraiz-Cardona I., Ortega E., Antón J.G., Pérez-Herranz V. (2011). Assessment of the roughness factor effect and the intrinsic catalytic activity for hydrogen evolution reaction on Ni-based electrodeposits. Int. J. Hydrogen Energy.

[27] Huang J. (2018). Diffusion impedance of electroactive materials, electrolytic solutions and porous electrodes: Warburg impedance and beyond. Electrochim. Acta.

[28] Van Haeverbeke M., Stock M., De Baets B. (2022). Equivalent Electrical Circuits and Their Use across Electrochemical Impedance Spectroscopy Application Domains. IEEE Access.

[29] Sun Y., Ling S., Zhao D., Liu J., Liu Z., Song J. (2020). Through-mask electrochemical micromachining of micro pillar arrays on aluminum. Surf. Coat. Technol..

[30] Li H.S., Wang G.Q., Li L.W., Gao C.P., Qu N.S., Zhu D. (2017). Through-mask electrochemical machining of hole arrays on molybdenum sheets. Int. J. Adv. Manuf. Technol..

[31] Baldhoff T., Nock V., Marshall A.T. (2017). Through-Mask Electrochemical Micromachining of Aluminum in Phosphoric Acid. J. Electrochem. Soc..

[32] Zhao H., Zhang Y., Chen C., Liu X., Wang G., Tang J. (2023). Investigation of through-mask electrochemical machining process using a rib-connected photoresist for microcone array with large height-to-diameter ratio. Precis. Eng..

[33] Wu Z., Wang S., Zhang Y., Song J., Xue B. (2023). Optimization of Process Parameters for Laser Cutting of AZ31B Magnesium Alloy Based on Orthogonal Experiment and BP Neural Network. Trans. Indian Inst. Met..

[34] Xia S., Lin R., Cui X., Shan J. (2016). The application of orthogonal test method in the parameters optimization of PEMFC under steady working condition. Int. J. Hydrogen Energy.

[35] Liang Z., Liao S., Wen Y., Liu X. (2017). Component parameter optimization of strengthen waterjet grinding slurry with the orthogonal-experiment-design-based ANFIS. Int. J. Adv. Manuf. Technol..

[36] Zhu H., Zhang Z., Xu J., Xu K., Ren Y. (2018). An experimental study of micro-machining of hydroxyapatite using an ultrashort picosecond laser. Precis. Eng..

